# MUC13 is a crucial player in YAP1-β-catenin survival complex mediated cancer metastasis

**DOI:** 10.3389/fmed.2025.1727498

**Published:** 2026-01-15

**Authors:** Shabnam Malik, Mohammed Sikander, Murali M. Yallapu, Subhash C. Chauhan

**Affiliations:** 1 Division of Cancer Immunology and Microbiology, Medicine and Oncology Integrated Service Unit, School of Medicine, University of Texas Rio Grande Valley, McAllen, TX, United States; 2 South Texas Center of Excellence in Cancer Research, School of Medicine, University of Texas Rio Grande Valley, McAllen, TX, United States

**Keywords:** chemoresistance, anoikis, metastasis, MUC13, Yap1, β-catenin

Colorectal cancer (CRC) is a one of most commonest cause of cancer related mortality worldwide, with 1.9 million new cases reported globally in 2022 and an estimated 150,000 new cases in the USA in 2024 (1). Despite significant advancements in diagnostic and therapeutic strategies, prognosis is still poor and long-term survival rate continues to be limited by advanced metastasis (2, 3). For instance, localized colorectal cancer demonstrates favorable prognoses with effective treatment modalities, achieving a 5-year survival up to 91%, whereas metastatic colorectal cancer (mCRC) is associated with a 5-year survival rate of approximately 13% (4). Metastasis is a multistage process involving the spread of tumor cells from the primary location following detachment from the extracellular matrix (ECM) or loss of cellular anchorage. In normal tissues, the loss of anchorage or detachment from the extracellular matrix triggers a form of programmed cell death known as anoikis, and the capacity of cells to evade this process is termed anoikis resistance (AR) (5). Only a small proportion (approximately 0.02%) of detached cells remain viable and are successful to establish a metastatic lesion (6). Metastasis is significantly slowed down by these anoikis after cells separate from the extracellular matrix (7–9). The detachment and dissemination of cancer cells from the extracellular matrix involve a number of critical steps that are essential for maintaining cancer cell viability (10). Additionally, it also encompasses the development of oncogenic phenotypes following their colonization of metastatic niches. Disruption at any level of cascade could hamper the formation of metastatic lesions, with AR being a critical step in the development of therapy (11, 12).

We, for the first time, have identified the pivotal significance of Mucin13 (MUC13), a transmembrane glycoprotein, in the process of cancer metastasis (13). MUC13 has demonstrated oncogenic potential in various cancer types, including colorectal carcinoma (14). MUC13 comprises three epidermal growth factor–like domains and a transmembrane domain and an active cytoplasmic tail. The cytoplasmic tail of MUC13 contains several potential phosphorylation sites (14–17). Our laboratory has demonstrated that MUC13 interacts functionally with and activates HER2 at the p1248 site, thereby triggering growth and survival signaling pathways such as FAK, ERK1/2, PAK1, and AKT. It also modulated metabolic reprograming through the activation and nuclear translocation of NF-κB p65, as well as the phosphorylation of IκB, thereby contributing to tumorigenesis (18, 19). Moreover, the expression of MUC13 has been associated with the advancement of malignancies, clinical prognosis, and biophysical alterations within cancerous cells (20, 21). Despite the established involvement of this factor in tumorigenesis, encompassing various signaling pathways, its specific role in the process of cancer metastasis remains incompletely understood (17, 22–28).

**Figure 1:**
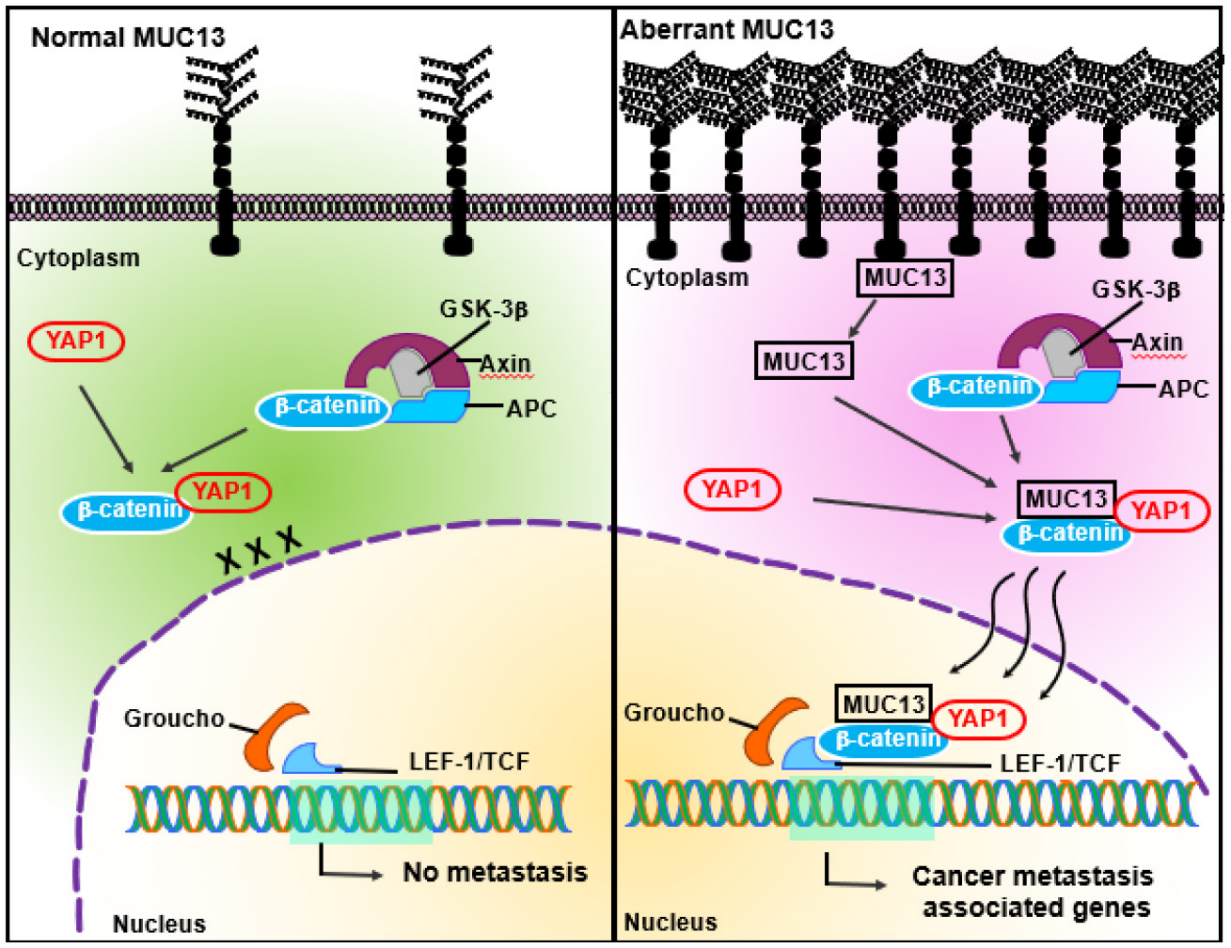
Simplified view of MUC13-mediated mechanism in metastatic colorectal cancer, highlighting the role of MUC13 in promoting the nuclear translocation of YPA1 and β-catenin.

Our research has demonstrated that MUC13 serves as a pivotal molecular determinant, promoting anchorage-independent survival and enhancing metastasis by modulating Yes-Associated Protein 1 (YAP1), a key transcriptional coactivator and regulator of the Hippo pathway (13) (Figure 1). Notably, we have demonstrated that MUC13 promotes metastasis primarily by modulating and inducing the nuclear translocation of YAP1, which leads to the activation and expression of genes involved in cell survival and metastatic progression (29–33). Abnormal expression of YAP1 has been observed in multiple malignancies including colorectal carcinoma especially *via* Hippo signaling pathway, governs numerous vital biological functions that underpin cellular viability, proliferation, differentiation, and organogenesis (29, 34, 35). Under normal condition, the Hippo–YAP1 signaling pathway is crucial for preserving cellular homeostasis in the colon, whereas YAP1 expression is elevated in colorectal cancers. The upregulation of YAP1 has been associated with increased cellular proliferation, improved cell survival, elevated drug resistance, induction of epithelial-to-mesenchymal transition, and anchorage-independent growth (29, 35). This novel molecular mechanism, mediated by MUC13-YAP1, provides a crucial survival benefit to anchorage-independent circulating tumor cells, thereby promoting successful extravasation, homing, and the aggressive metastasis of cancer to distant sites.

To investigate the role of MUC13 in promoting anchorage-independent survival in cancer cells, we developed and validated an anoikis induction model (13). Stable cell lines SW480 cells overexpressing MUC13 and SW620 cells with MUC13 knockdown were generated. Our experimental findings indicate MUC13 contributes significantly to cancer cell survival under anchorage-independent environment and facilitates AR. Proteomics and kinome array analyses were conducted to elucidate the specific molecular mechanisms by which MUC13 confers protection against cell mortality in anchorage-independent conditions. These assays indicated the differential expression of multiple genes associated with survival and metastasis. Proximity ligation assay (PLA) and Immunoprecipitation (IP) assays indicated a novel molecular interaction between MUC13 and YAP1. Studies have indicated a cooperative interaction between YAP1 and β-catenin in the survival of cancer cells through the upregulation of oncogenic effector proteins (36–40). β-catenin functions as a transcription factor that modulates the Wnt signaling pathway, reported to have a crucial role in the development of CRC (39, 41, 42). Consequently, an investigation into the impact of MUC13 on the YAP1/β-catenin survival complex was performed. Ectopic expression of MUC13 promoted the nuclear translocation and localization of the YAP1/β-catenin survival complex. Notably, the inhibition of MUC13 prevented the integration of this survival complex into the nucleus, as it was predominantly localized at the periphery of the nuclear envelope, and it regulated the expression of downstream genes linked to survival and metastasis.

The ability of cancer cells to survive in the circulatory system and subsequently invade distal sites is a critical characteristic of metastasis. To investigate the impact of MUC13-mediated nuclear translocation of the survival complex (YAP1–β-catenin) on metastatic potential, a tail vein metastasis model was used. It was demonstrated that ectopic MUC13 expression provided a metastatic advantage to SW480/MUC13 cells, as indicated by the presence of metastatic lesions in the kidneys, lungs, and liver. Conversely, no metastatic lesions were detected in SW480+vector control cells. High expression of MUC13, YAP1, and β-catenin was observed in metastatic lesions of the lung and kidney. These findings indicate that MUC13 plays a vital role in promoting YAP1-driven oncogenic and metastatic signaling mechanisms within malignant cells. Numerous studies have demonstrated that mucins are crucial for anoikis resistance by aiding in detachment from the extracellular matrix and promoting the survival of tumor cells (43–46). This underscores the concept that MUC13 plays a pivotal role in promoting cancer progression and metastasis *via* the YAP1-dependent oncogenic pathway. While this study establishes a firm foundation for comprehending the function of MUC13 in the metastasis of colorectal cancer, several unresolved questions necessitate further investigation. For instance, it is essential to examine the precise mechanism by which MUC13 facilitates the nuclear translocation of YAP1.

Therefore, it is essential to examine whether MUC13, in addition to conferring resistance to anoikis in cancer cells, can also promote subsequent stages of the metastatic process. Are additional co-factors implicated in the MUC13-YAP1 interaction? The influence of the tumor microenvironment on modulating MUC13 expression, and the subsequent effects on YAP1 activity, introduces an additional level of intricacy that warrants further investigation. Last but not least, it would be valuable to investigate how the extracellular matrix components that encircle stromal and immune cells influence the function of MUC13. Specifically, small molecules and/or monoclonal antibodies designed to inhibit MUC13 could potentially interfere with its interaction with YAP1, thereby diminishing the activation of metastasis-related genes. Complementing therapies that directly target MUC13 with interventions aimed at these downstream effectors could facilitate the development of a multimodal strategy for disrupting the metastatic cascade.

Consequently, the biochemical modulation of this novel molecular interaction holds the potential to specifically address and treat cancer metastasis.
